# Beta-blockers after acute coronary syndrome: insights across the ejection fraction spectrum

**DOI:** 10.1093/ehjopen/oeag175

**Published:** 2026-10-14

**Authors:** Eirinaios Tsiartas, Sokratis Oikonomou, Aristea Iordanidou, Athanasios Samaras, Andreas S Papazoglou, Dimitrios V Moysidis, Vassileios Vassilikos, Antonios Ziakas, Periklis Davlouros, Konstantinos Tsioufis, Dimitrios N Tziakas, Ioannis Zarifis, George Giannakoulas

**Affiliations:** Medical Division, Addenbrooke's Hospital, Cambridge University Hospitals NHS Foundation Trust, Hills Road, Cambridge CB2 0QQ, UK; School of Medicine, Aristotle University of Thessaloniki, University Campus, Thessaloniki 541 24, Greece; School of Medicine, Aristotle University of Thessaloniki, University Campus, Thessaloniki 541 24, Greece; School of Medicine, Aristotle University of Thessaloniki, University Campus, Thessaloniki 541 24, Greece; Second Cardiology Department, Hippokrateion University Hospital, Aristotle University of Thessaloniki, 49 Konstantinoupoleos, Thessaloniki 54642, Greece; Department of Cardiology, Athens Naval Hospital, 70 Dinokratous, Athens 115 21, Greece; School of Medicine, Aristotle University of Thessaloniki, University Campus, Thessaloniki 541 24, Greece; Third Cardiology Department, Hippokrateion University Hospital, Aristotle University of Thessaloniki, 49 Konstantinoupoleos, Thessaloniki 54642, Greece; First Department of Cardiology, AHEPA University Hospital, School of Medicine, Faculty of Health Sciences, Aristotle University of Thessaloniki, 1 Kiriakidi, Thessaloniki 546 36, Greece; Department of Cardiology, Faculty of Medicine, School of Health Sciences, University Hospital of Patras, Hippocrates Street (University Campus), Rio Patras 265 04, Greece; First Cardiology Department, Hippokration General Hospital, University of Athens Medical School, 114 Vasilissis Sofias, Athens 115 27, Greece; Department of Cardiology, Medical School, Democritus University of Thrace, University Hospital of Alexandroupolis, Alexandroupolis 681 00, Greece; Department of Cardiology, ‘George Papanikolaou’ General Hospital, Leoforos Papanikolaou, Exochi 57010, Thessaloniki, Greece; First Department of Cardiology, AHEPA University Hospital, School of Medicine, Faculty of Health Sciences, Aristotle University of Thessaloniki, 1 Kiriakidi, Thessaloniki 546 36, Greece

**Keywords:** Acute coronary syndrome, Myocardial infarction, Beta-blocker, Left ventricular ejection fraction, Secondary prevention

## Abstract

**Aims:**

To evaluate the association between beta-blocker (BB) prescription and long-term all-cause mortality after acute coronary syndrome (ACS), and to assess whether this varies across the left ventricular ejection fraction (LVEF) spectrum.

**Methods and results:**

We analysed ACS patients from the retrospective multicentre CardioMining study (NCT05176769). The primary analysis used inverse probability of treatment weighting (IPTW) and restricted mean survival time (RMST) derived from weighted Kaplan–Meier curves. RMST and survival differences were estimated at 1 and 5 years. A landmark analysis evaluated survival between years 1 and 5 among first-year survivors. Among 6049 ACS patients (age 66.8 ± 13.2 years; 74.9% male), 5107 (84.4%) were discharged on BBs. During a median 4.6-year follow-up, 1912 deaths (31.6%) occurred. Overall, the 1-year RMST difference associated with BBs was 6.3 days (95%CI 0.0 to 13.2), which attenuated further by 5 years (11.3 days, 95%CI −30.7 to 63.9). The largest 1-year RMST difference was observed among patients with LVEF ≤40% (18.8 days, 95%CI 1.1 to 37.4), whereas associations were smaller and non-significant among patients with LVEF 41–49% and LVEF ≥50%. At 5 years, confidence intervals crossed the null across all LVEF categories. Continuous LVEF analyses suggested greater short-term benefit at lower values, with progressive attenuation across the LVEF spectrum. Among 1-year survivors, no significant RMST differences were observed between years 1 and 5.

**Conclusion:**

Discharge BB prescription was not associated with a clear long-term survival advantage after ACS in this large real-world cohort. Apparent associations were strongest at lower LVEF values and attenuated during longer follow-up.

## Graphical Abstract

**Graphical Abstract:**
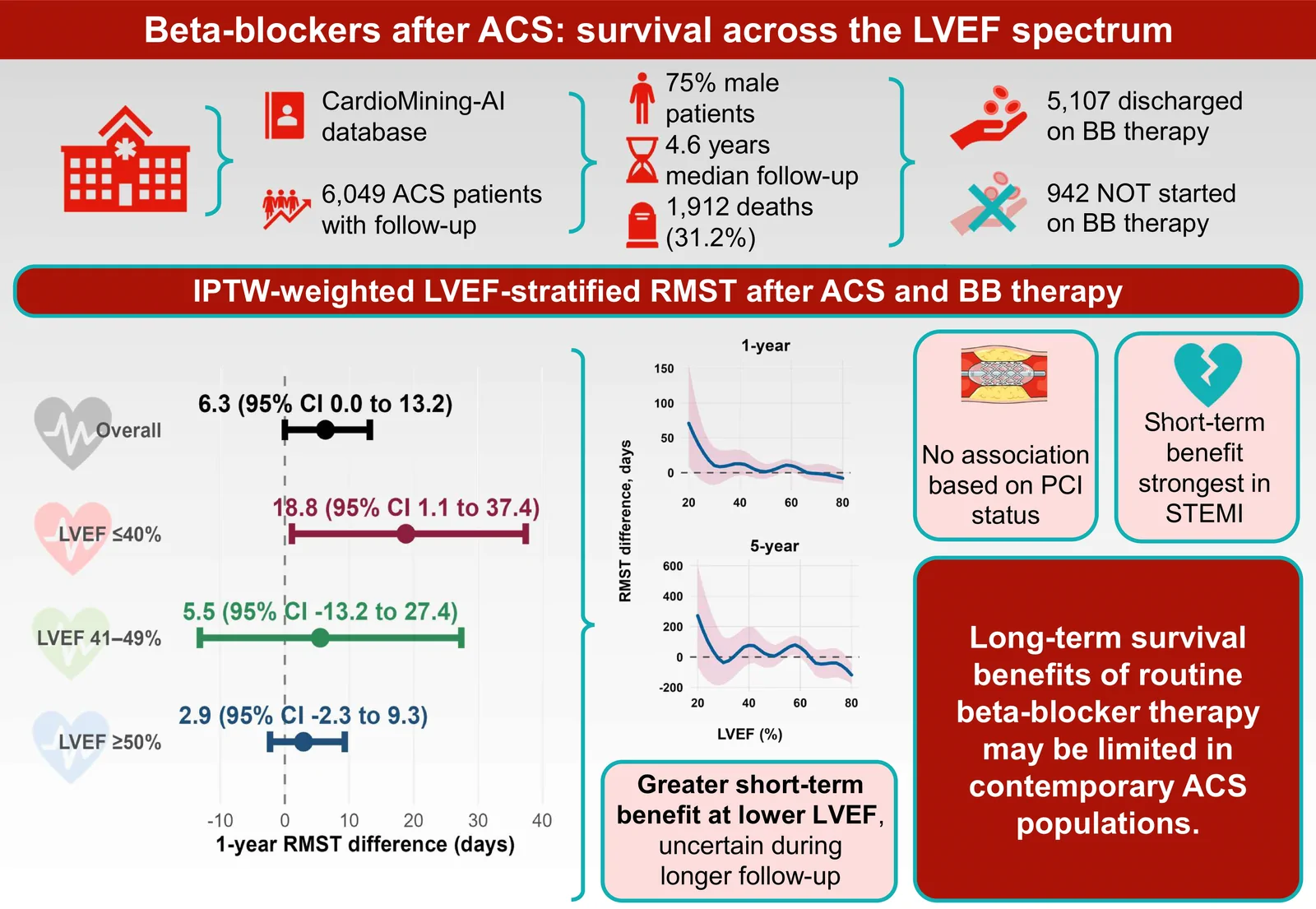

Key findings

- In over 6000 patients treated for ACS across multiple hospitals, long-term survival was similar between patients prescribed or not prescribed a BB at discharge after accounting for differences in age, comorbidities, heart function, and treatment.
- The largest survival differences were observed during the first year after discharge among patients with reduced heart-pumping function (left ventricular ejection fraction ≤40%), but these differences became less apparent over longer follow-up. Among patients who survived the first year after discharge, no clear survival differences were observed thereafter.

## Introduction

Beta-blockers (BBs) have long been considered a cornerstone of secondary prevention after acute coronary syndrome (ACS).^1–4^ However, their benefits were primarily established in the pre-reperfusion era, before the widespread adoption of thrombolysis and percutaneous coronary intervention (PCI). Additionally, contemporary ACS management, including modern pharmacotherapy, has substantially improved outcomes, potentially reducing the incremental benefit of BB therapy.^5,6^

Against this background, the left ventricular (LV) ejection fraction (LVEF) has emerged as a key modifier of treatment benefit. The efficacy of BBs is more established in patients with reduced LVEF, in whom their use has consistently been shown to reduce mortality and morbidity.^1–3,5,6^ In contrast, evidence among patients with LVEF >40% remains less consistent.^6–13^ Recent randomized controlled trials (RCTs) have shown no significant reduction in all-cause mortality, reinfarction, or heart failure (HF) hospitalization with routine post-ACS BB therapy in patients with preserved LVEF.^8,14^ Consequently, there is a growing need for pragmatic data reflecting contemporary clinical practice across the spectrum of LV systolic function.

In this study, we examined the association between discharge BB therapy and all-cause mortality among patients hospitalized with ACS, drawing on data from a multicentre real-world registry. We further evaluated this association across the LVEF spectrum and its temporal pattern following discharge to determine whether any apparent survival associations varied according to the degree of LV systolic dysfunction and the timing after discharge (*Graphical Abstract*).

## Methods

### Study design

This retrospective observational cohort study used data from the CardioMining database and is reported in accordance with the STROBE guidelines (see Supplementary material online, *Table S1*).^15^ CardioMining (ClinicalTrials.gov identifier: NCT05176769) is an ongoing multicentre study of cardiovascular hospitalization data in Greece. The registry combines manually extracted clinical information from electronic health records with prescribing and mortality data obtained through the Greek Ministry of Health. The detailed study protocol has been published previously.^16^ The study was approved by the Medical Research Ethics Committee of the Aristotle University of Thessaloniki (Reference: 19513/24.5.2021) and the institutional ethics boards of all participating hospitals. The requirement for informed consent was waived owing to the retrospective nature of the study.

### Population, data collection, and outcome of interest

The current version of the CardioMining database comprises quality-checked, manually extracted data from 46 305 cardiovascular hospitalizations among patients discharged alive from seven Greek hospitals between 2010 and 2023. Participating centres contributed data for different calendar periods and with varying availability of mortality follow-up, reflecting the progressive implementation of linkage with national mortality and electronic prescribing platforms (see Supplementary material online, *Table S2*).

We included patients admitted with ACS who had available follow-up data, LVEF recorded during index hospitalization, and discharge BB prescription status. Missing LVEF values largely reflected limitations in the availability of electronically accessible echocardiographic reports across centres. All patients included in the primary cohort had complete data for variables used in the propensity score model. Left ventricular ejection fraction was categorized into three clinically relevant groups: preserved (≥50%), mildly reduced (41–49%), and reduced (≤40%). The primary exposure was prescription of an oral BB at hospital discharge. The primary outcome was all-cause mortality during follow-up.

### Statistical analysis

Baseline characteristics were summarized using means with standard deviations (SDs) for continuous variables and counts with percentages for categorical variables.

#### Propensity score weighting

To minimize treatment-selection bias, inverse probability of treatment weighting (IPTW) was performed using a propensity score model estimating the probability of discharge BB prescription. The propensity score model included age, sex, ACS subtype, PCI during the index admission, LVEF, previous coronary artery disease (CAD), HF, hypertension, type 2 diabetes mellitus (T2DM), chronic kidney disease (CKD), chronic obstructive pulmonary disease (COPD), and atrial fibrillation (AF). Stabilized average treatment effect weights were truncated at the 1st and 99th percentiles to reduce the influence of extreme observations.

#### Primary outcome analysis

The primary analysis assessed the association between discharge BB prescription and survival using IPTW-weighted restricted mean survival time (RMST) estimated from weighted Kaplan–Meier curves. Restricted mean survival time and survival probabilities were estimated at 1 and 5 years and expressed as absolute differences between patients discharged with and without BB. Confidence intervals (CIs) were obtained using non-parametric bootstrap resampling.

As a supportive analysis, an IPTW-weighted Cox proportional hazards model including discharge BB prescription, LVEF category, and their interaction was fitted. Hazard ratios (HRs) with 95%CIs were reported. The proportional hazards (PH) assumption was assessed using Schoenfeld residuals. Given the limitations of HR-based inference when hazards are non-proportional, Cox model estimates were considered supportive rather than primary measures of association.

#### Additional analyses

Prespecified subgroup analyses evaluated whether associations differed according to LVEF category (≤40%, 41–49%, and ≥50%), PCI status, and ACS subtype (ST-elevation myocardial infarction, non-ST-elevation myocardial infarction, and unstable angina). A landmark analysis was also performed among patients surviving the first year after discharge to evaluate associations between BB prescription and subsequent survival between years 1 and 5.

Associations were also examined across the continuous LVEF spectrum using centred overlapping LVEF windows. A permutation-based dependency test assessed whether RMST differences varied across LVEF values.

As a sensitivity analysis, we compared patients with persistent BB use and persistent non-use among those with available follow-up dispensed prescriptions data.

All analyses were conducted using R version 4.5.1 (R Foundation for Statistical Computing, Vienna, Austria). Statistical significance was defined as a two-sided *P*-value <0.05.

## Results

### Patient characteristics

Of 46 305 cardiovascular hospitalizations in the CardioMining database, 6049 met the inclusion criteria (*Figure 1*). The cohort comprised 2165 (35.8%) patients with STEMI, 2304 (38.1%) with NSTEMI, and 1580 (26.1%) with unstable angina. ACS management according to presentation subtype is presented in Supplementary material online, *Table S3*. During a median follow-up of 4.6 years (IQR 2.4–6.5), 1912 deaths (31.6%) were recorded.

**Figure 1:**
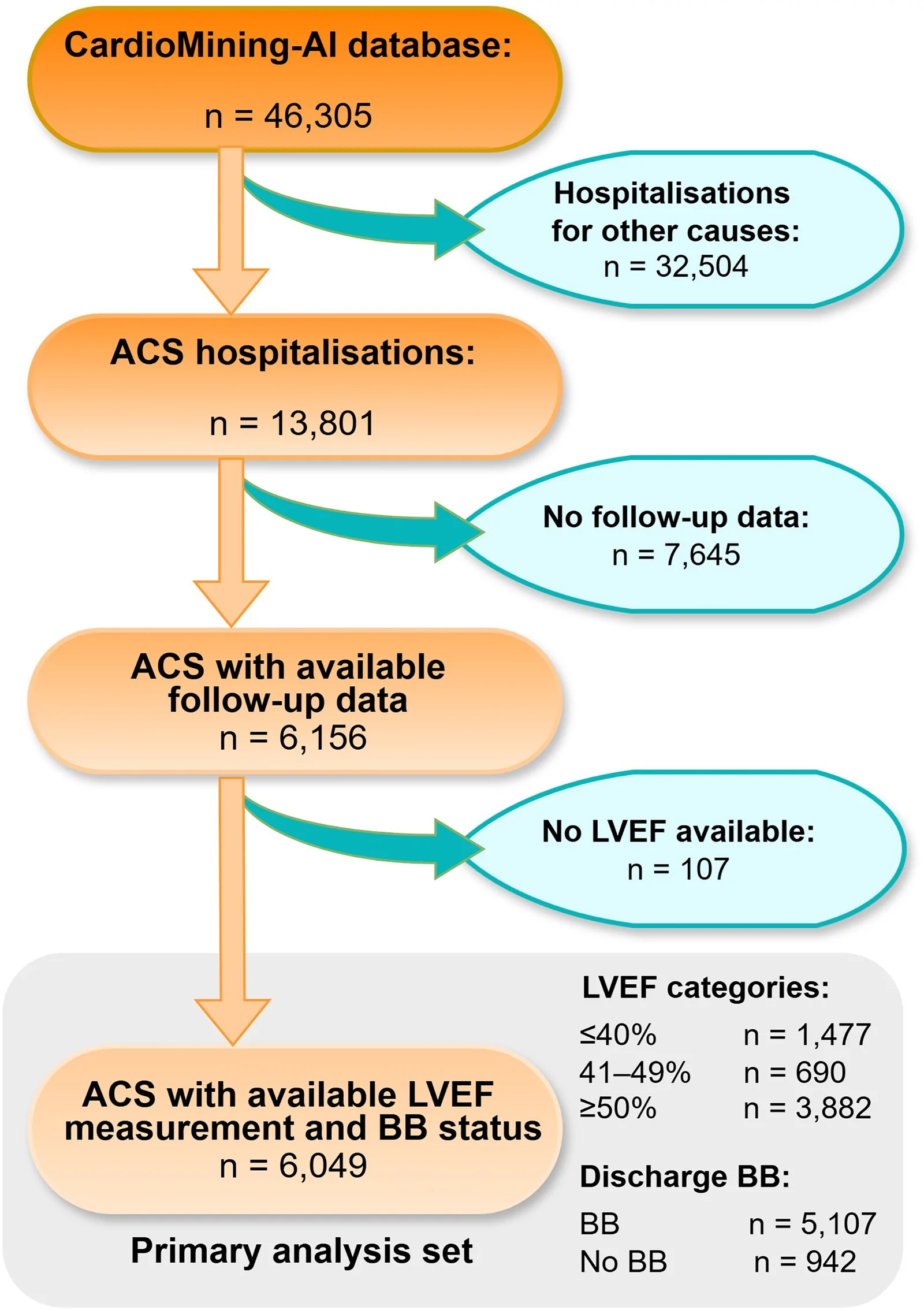
Study flowchart of patient selection from the CardioMining study. ACS, acute coronary syndrome; BB, beta-blocker; LVEF, left ventricular ejection fraction.

At discharge, 5107 patients (84.4%) received a BB, while 942 (15.6%) did not. The most common agent across all LVEF groups was bisoprolol (*n* = 2239; 43.8%) (see Supplementary material online, *Table S4*). Compared with patients not prescribed BB, those discharged on BB were younger (66.4 ± 13.1 vs. 68.8 ± 13.6 years; *P* < 0.001), more likely to undergo PCI during the index admission (49.8% vs. 40.6%; *P* < 0.001), less likely to have a history of atrial fibrillation (8.6% vs. 12.0%; *P* = 0.001), and more likely to have LVEF ≤40% (25.0% vs. 21.2%; *P* < 0.001) (*Table 1*).

**Table 1:** Baseline characteristics of included patients based on beta-blocker status at discharge

	Overall (*n* = 6049)	No BB (*n* = 942)	BB (*n* = 5107)	*P* value
Age, years	66.8 ± 13.2	68.8 ± 13.6	66.4 ± 13.1	<0.001
Male sex	4533 (74.9%)	690 (73.2%)	3843 (75.2%)	0.207
Follow-up duration, years	4.6 (2.4–6.5)	4.7 (2.3–6.9)	4.5 (2.4–6.4)	0.068
All-cause deaths	1912 (31.6%)	315 (33.4%)	1597 (31.3%)	0.202
ACS presentation				
STEMI	2165 (35.8%)	310 (32.9%)	1855 (36.3%)	0.023
NSTEMI	2304 (38.1%)	354 (37.6%)	1950 (38.2%)	
Unstable angina	1580 (26.1%)	278 (29.5%)	1302 (25.5%)	
PCI during admission	2925 (48.4%)	382 (40.6%)	2543 (49.8%)	<0.001
LVEF				
LVEF (numerical)	54.0 ± 16.1	55.8 ± 16.2	53.7 ± 16.1	<0.001
LVEF ≤40%	1477 (24.4%)	200 (21.2%)	1277 (25.0%)	<0.001
LVEF 41–49%	690 (11.4%)	80 (8.5%)	610 (11.9%)	
LVEF ≥50%	3882 (64.2%)	662 (70.3%)	3220 (63.1%)	
Prior history				
Previous CAD	2535 (41.9%)	404 (42.9%)	2131 (41.7%)	0.530
Previous heart failure	459 (7.6%)	72 (7.6%)	387 (7.6%)	0.998
Hypertension	2836 (46.9%)	425 (45.1%)	2411 (47.2%)	0.251
Type 2 diabetes mellitus	1765 (29.2%)	244 (25.9%)	1521 (29.8%)	0.018
Chronic kidney disease	548 (9.1%)	82 (8.7%)	466 (9.1%)	0.726
Chronic obstructive pulmonary disease	263 (4.3%)	44 (4.7%)	219 (4.3%)	0.658
Atrial fibrillation	551 (9.1%)	113 (12.0%)	438 (8.6%)	0.001

ACS, acute coronary syndrome; BB, beta blocker; CAD, coronary artery disease; LVEF, left ventricular ejection fraction; NSTEMI, non-ST elevation myocardial infarction; PCI, percutaneous coronary intervention; STEMI, ST elevation myocardial infarction.

Overall, 1477 patients (24.4%) had LVEF ≤40%, 690 (11.4%) had LVEF 41–49%, and 3882 (64.2%) had LVEF ≥50%, with BB prescription rates exceeding 80% across all LVEF categories. Baseline characteristics of included and excluded patients were comparable (see Supplementary material online, *Table S5*). Following IPTW, all covariates achieved adequate balance (see Supplementary material online, *Figure S1*; model diagnostics in Supplementary material online, *Table S6*). Baseline characteristics in the weighted pseudo-population are presented in Supplementary material online, *Table S7*.

### Association between discharge BB prescription and survival

IPTW-weighted Kaplan–Meier curves demonstrated largely overlapping survival trajectories between patients discharged with and without BBs throughout follow-up (*Figure 2*).

**Figure 2:**
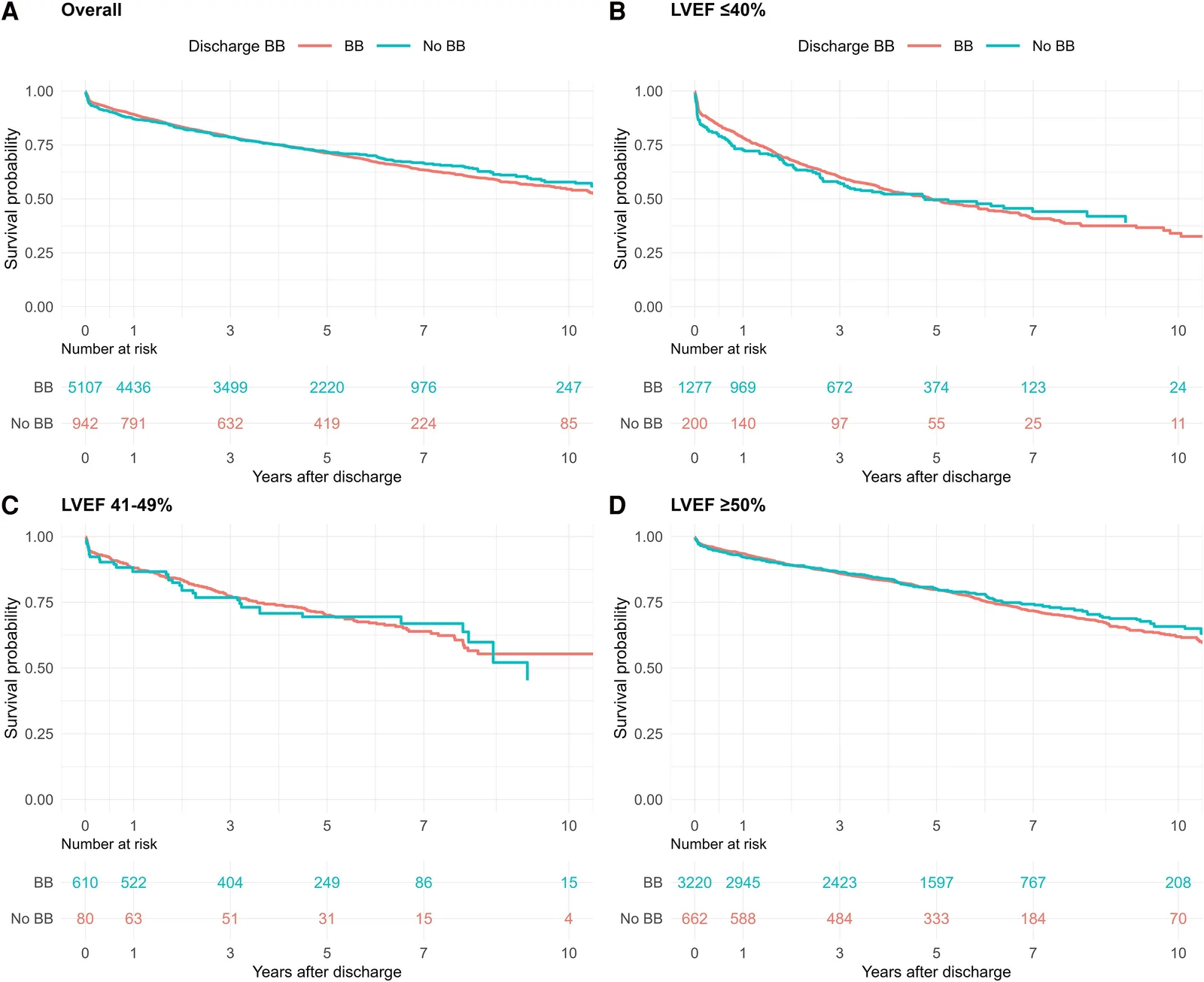
IPTW-weighted Kaplan–Meier survival according to discharge beta-blocker prescription overall (*A*) and by left ventricular ejection fraction category: ≤40% (*B*), 41–49% (*C*), and ≥50% (*D*). BB, beta-blocker; IPTW, inverse probability of treatment weighting; LVEF, left ventricular ejection fraction.

At 1 year, discharge BB prescription was associated with a modest absolute increase in RMST of 6.3 days (95%CI 0.0 to 13.2) (*Figure 3*, Supplementary material online, *Table S8*) and an absolute survival difference of 2.1% (95%CI −0.1% to 4.2%). However, CIs widened over longer follow-up, and no statistically significant differences were observed at 5 years (RMST difference 11.3 days, 95%CI −30.7 to 63.9; absolute survival difference −0.5%, 95%CI −3.4% to 3.2%).

**Figure 3:**
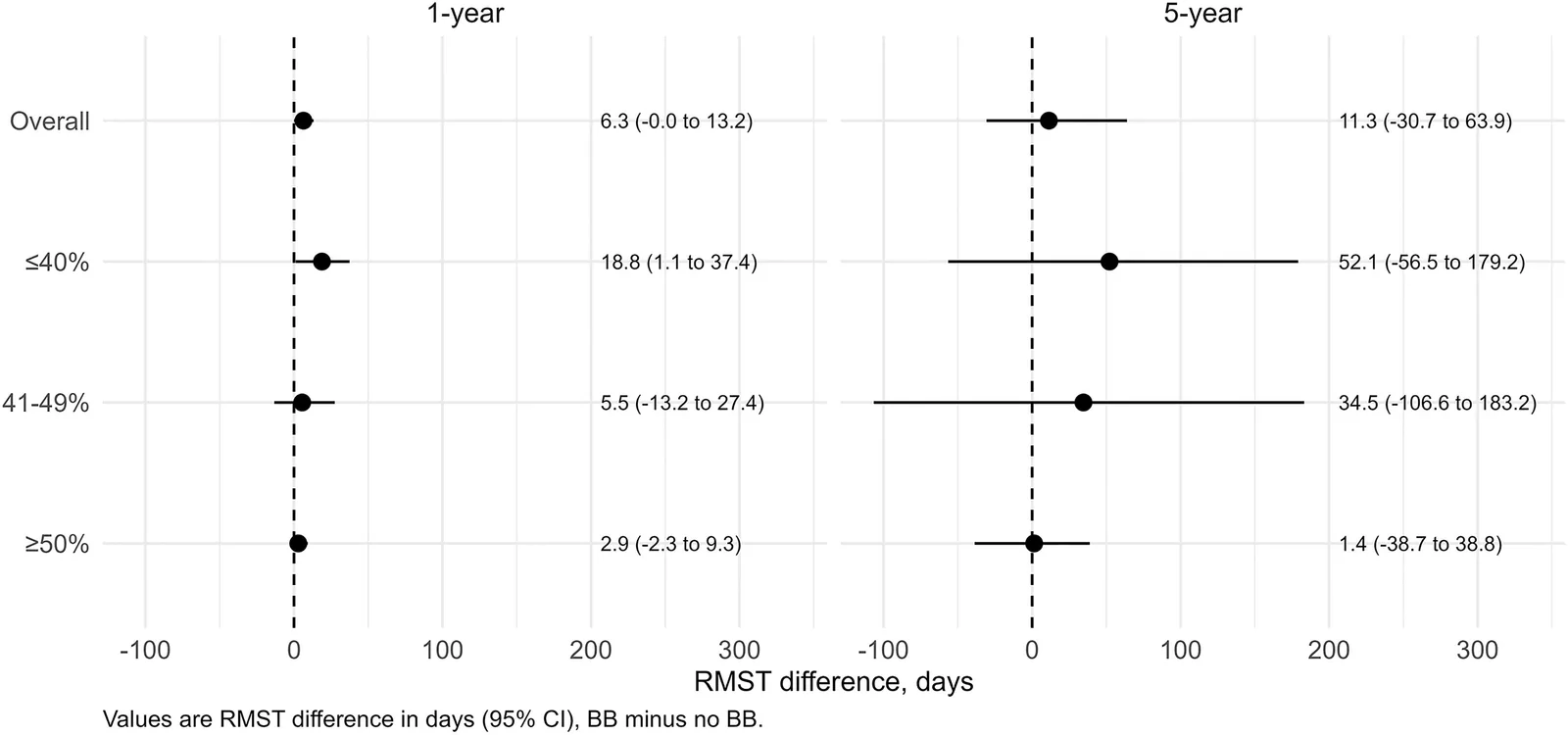
Restricted mean survival time differences associated with discharge beta-blocker prescription across left ventricular ejection fraction categories at 1 (left) and 5 years (right). BB, beta-blocker; CI, confidence interval; RMST, restricted mean survival time.

### Association according to left ventricular ejection fraction category

At 1 year, patients with LVEF ≤40% experienced the largest RMST difference associated with BB prescription (18.8 days; 95%CI 1.1 to 37.4) and an absolute survival difference of 5.5% (95%CI 0.0% to 11.9%) (*Figures 2* and *3*). In contrast, smaller and non-significant differences were observed among patients with LVEF 41–49% (RMST difference 5.5 days, 95%CI −13.2 to 27.4) and those with LVEF ≥50% (RMST difference 2.9 days, 95%CI −2.3 to 9.3). At 5 years, RMST differences were numerically greater among patients with LVEF≤40% (52.1 days) and LVEF 41–49% (34.5 days) than among those with LVEF ≥50% (1.4 days), although CIs crossed the null for all groups. Corresponding survival differences were small and non-significant (see Supplementary material online, *Table S8*).

In a landmark analysis restricted to patients surviving the first year after discharge, no significant survival differences were observed between years 1 and 5 in the overall cohort or within any LVEF category (*Table 2*).

**Table 2:** Landmark analysis of survival from year 1 to year 5 according to discharge beta-blocker prescription

Subgroup	No BB (*n*)	BB (*n*)	RMST difference, days (95%CI)	Survival difference, % (95%CI)
Overall	791	4436	−26.0 (−51.0 to 0.1)	−2.5 (−5.2 to 0.7)
LVEF ≥50%	588	2945	−21.1 (−44.5 to 3.6)	−1.4 (−4.1 to 2.0)
LVEF 41–49%	63	522	10.5 (−77.7 to 104.0)	−0.7 (−10.7 to 10.7)
LVEF ≤40%	140	969	−41.7 (−118.6 to 46.6)	−5.2 (−14.3 to 3.2)

BB, beta-blocker; CI, confidence interval; LVEF, left ventricular ejection fraction; RMST, restricted mean survival time.

Note: Landmark analyses were restricted to patients surviving the first year following discharge. RMST and survival differences were calculated between years 1 and 5.

In the supportive IPTW-weighted Cox model, discharge BB prescription was not associated with mortality in patients with LVEF ≤40% (HR 1.07, 95%CI 0.83–1.38), and there was no evidence of differing association according to LVEF category (*P*-interaction=0.967) (see Supplementary material online, *Table S9*). However, the proportional hazards assumption was violated globally (*P* < 0.001) (see Supplementary material online, *Table S10*), supporting the use of RMST as the primary estimand. Unweighted analyses yielded larger effect estimates than IPTW-weighted analyses, consistent with residual confounding in crude comparisons (see Supplementary material online, *Table S11*).

### Association according to PCI status and ACS subtype

There was no clear evidence that the association between BB prescription and RMST differed according to PCI status (*Figure 4*, Supplementary material online, *Table S12*). When stratified by ACS subtype, the largest short-term association was observed among STEMI patients. At 1 year, BB prescription was associated with an RMST difference of 11.3 days (95%CI 4.0 to 22.2) and an absolute survival difference of 2.5% (95%CI 0.3% to 5.6%). However, the corresponding 5-year RMST difference was smaller and no longer statistically significant (26.8 days, 95%CI −20.6 to 80.3). Among patients with NSTEMI and unstable angina, RMST differences were small and not significant.

**Figure 4:**
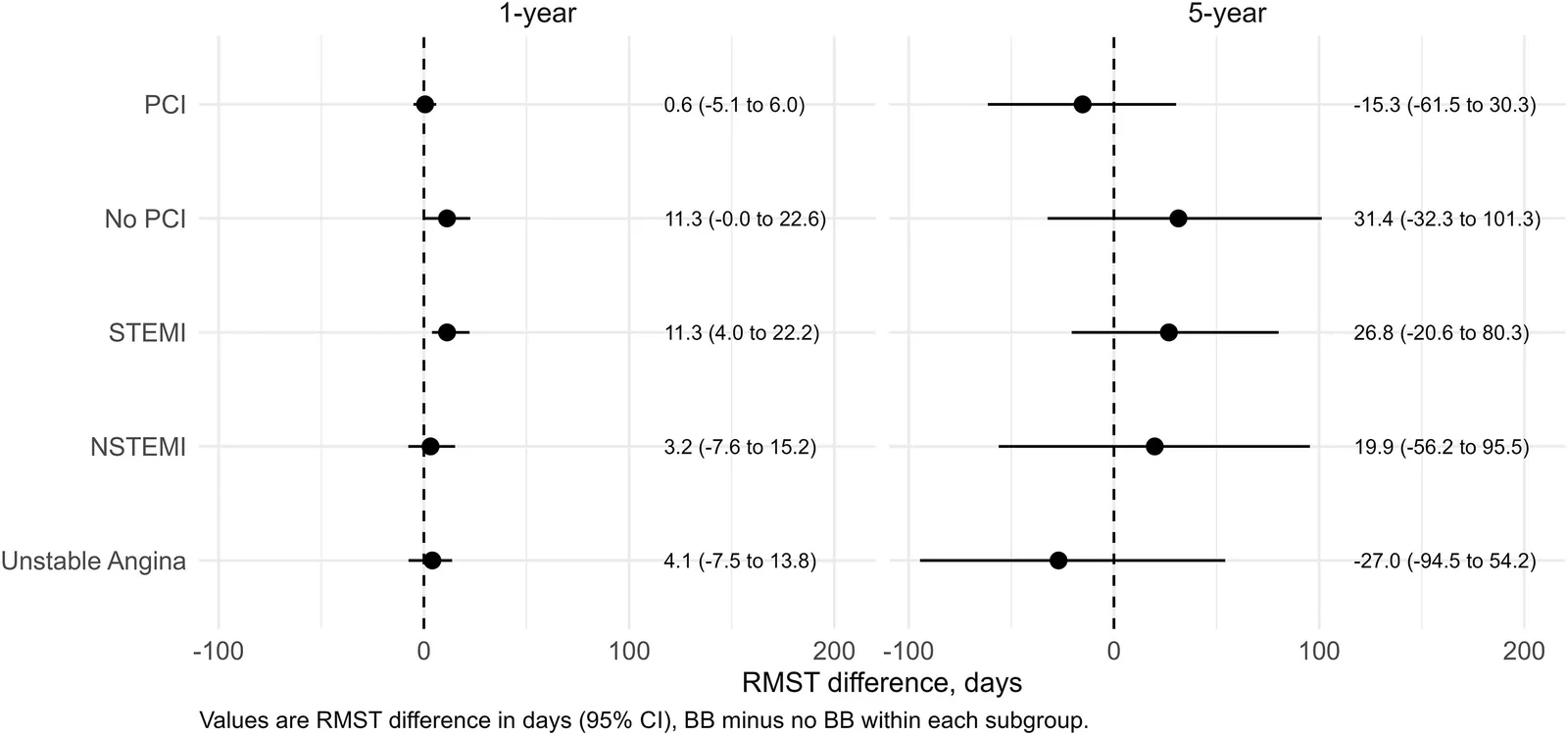
Restricted mean survival time differences associated with discharge beta-blocker prescription according to PCI status and acute coronary syndrome subtype at 1 (left) and 5 years (right). BB, beta-blocker; CI, confidence interval; NSTEMI, non-ST-elevation myocardial infarction; PCI, percutaneous coronary intervention; RMST, restricted mean survival time; STEMI, ST-elevation myocardial infarction.

### Analysis across the continuous left ventricular ejection fraction spectrum

At 1 year, positive RMST differences were most apparent at lower LVEF values and tended to attenuate with increasing LVEF (dependency *P* < 0.001), approaching the null and becoming slightly negative at higher LVEF values. Confidence intervals crossed the null across much of the LVEF spectrum. At 5 years, RMST differences remained dependent on LVEF (dependency *P* = 0.002), with generally positive estimates at lower and intermediate LVEF values and negative estimates at higher LVEF values. However, CIs crossed the null even at reduced LVEF (*Figure 5*).

**Figure 5:**
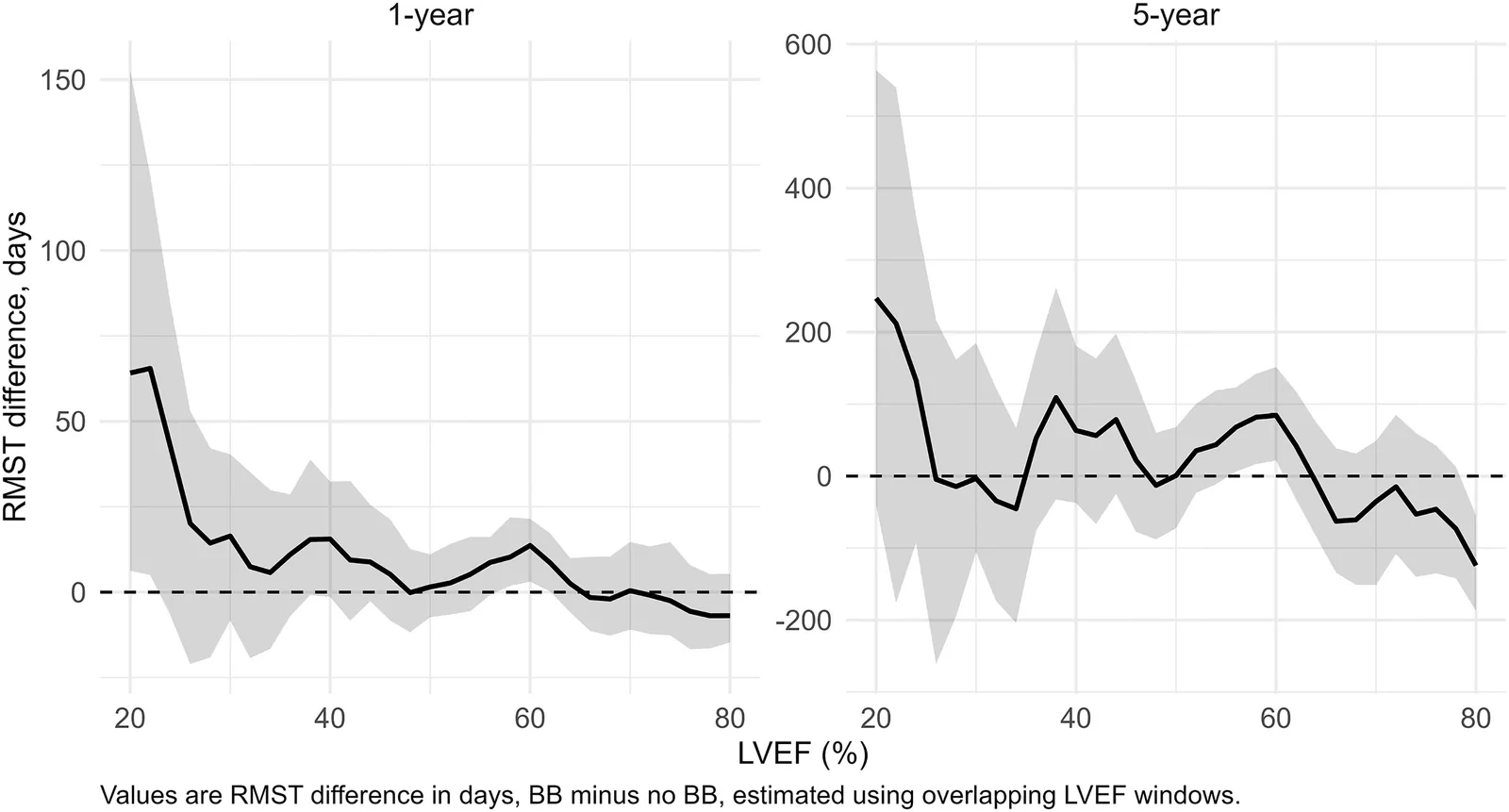
Restricted mean survival time differences associated with discharge beta-blocker prescription across the entire left ventricular ejection fraction spectrum at 1(left) and 5 years (right) with shaded areas denoting 95% confidence intervals. BB, beta-blocker; LVEF, left ventricular ejection fraction; RMST, restricted mean survival time.

### Persistent BB prescription/non-prescription sensitivity analysis

Follow-up prescription data were available for 6039 patients. Analyses restricted to patients with persistent BB prescriptions (*n* = 3993) or persistent non-use (*n* = 556) yielded results consistent with the primary analysis (see Supplementary material online, *Table S13*). Consistent with the primary analysis, the largest RMST differences were observed among patients with LVEF ≤40% and LVEF 41–49%, although CIs remained wide and included the null value.

## Discussion

In this large multicentre real-world cohort of ACS patients, BB prescription at discharge was not associated with a clear reduction in all-cause mortality. Overall RMST differences were small and attenuated over time, with no meaningful survival differences observed at 5 years. Although the largest short-term RMST differences were observed among patients with LVEF ≤40%, uncertainty around subgroup estimates remained substantial, and these differences were less apparent at 5 years. Continuous LVEF analyses similarly suggested greater short-term benefit at lower LVEF values, with progressive attenuation across the LVEF spectrum. Taken together, these findings suggest that any survival advantage associated with BB therapy in contemporary ACS populations is likely modest, may be concentrated among patients with impaired LV systolic function, and may be largely confined to the early post-discharge period.

Current international guidelines generally continue to support routine BB therapy after ACS regardless of LVEF.^17,18^ In contrast, the 2025 Australian National Heart Foundation guideline takes a more nuanced position advising that BBs may be withheld in revascularized ACS patients with preserved LV systolic function on optimal medical therapy.^19^ Rapid reperfusion and evidence-based pharmacotherapy reduce arrhythmic and remodelling risk, narrowing the window in which BBs can provide additional benefit.^5,20^ The observation that any apparent survival advantage was greatest during the first year after discharge and attenuated thereafter is compatible with a treatment effect that is strongest during the early post-ACS period, when sympathetic activation and arrhythmic vulnerability are most pronounced.

The evidence supporting post-ACS BB therapy has evolved considerably over time. Early pre-revascularisation trials reported mortality reductions and established the historical rationale for routine long-term BB therapy.^1–4^ Recent RCTs have yielded heterogeneous findings. Both REBOOT-CNIC and REDUCE-AMI reported neutral effects of sustained BB therapy in patients without reduced LVEF.^8,14^ BETAMI/DANBLOCK introduced additional complexity, by identifying potential benefit for certain composite endpoints in populations that included patients with mildly reduced LVEF. These differences may reflect variability in trial populations, BB agents, dosing strategies, background therapies, and event rates.^21^ Observational data have similarly reported inconsistency, with signals of benefit largely confined to patients with reduced LVEF.^6^

Our findings are broadly consistent with the most recent meta-analyses of contemporary randomized trials, which have not demonstrated a significant prognostic benefit of routine BB therapy in preserved LVEF.^10,12,13^ The principal contribution of the present study is not to challenge these data, but to extend them to a broad, pragmatic cohort spanning the entire LVEF spectrum and characterized by older age and greater comorbidity burden than contemporary trial populations. In contrast to recent trials that primarily focused on preserved or near-preserved LV systolic function, our analyses incorporated both categorical and continuous assessments of LVEF, allowing exploration of treatment associations across a wider range of LV function. Although CIs remained wide, the largest short-term RMST differences were observed among patients with reduced LVEF, while continuous LVEF analyses suggested progressive attenuation of any apparent benefit with increasing LVEF. Furthermore, the observation that differences were most evident during the first year after discharge but diminished thereafter is consistent with the hypothesis that any incremental benefit of BB therapy may be concentrated in the early post-ACS period. This interpretation is supported by recent evidence from SMART-DECISION, in which discontinuation of BB therapy after 1 year was non-inferior to continuation among stable post-myocardial infarction patients without LV systolic dysfunction.^22^ Collectively, these findings suggest that the timing, duration, and patient selection for BB therapy may be at least as important as the decision to prescribe BB therapy itself.

This study has several limitations. As an observational, non-randomized analysis, residual confounding by indication cannot be completely excluded. Specific contraindications to BB therapy and markers of acute haemodynamic status were unavailable and could not be incorporated into the propensity score model, although IPTW achieved excellent covariate balance. The analysis was restricted to patients discharged alive and therefore does not capture potential benefits of BB therapy during the early in-hospital phase of ACS, when anti-arrhythmic effects may be most relevant. Furthermore, all-cause mortality was the only available outcome. Any beneficial effects on cardiovascular or arrhythmic death may therefore have been attenuated by competing non-cardiovascular mortality. Left ventricular ejection fraction was measured during the index admission and recovery following revascularization may have resulted in some misclassification of LVEF categories. Information on BB dosage and medication adherence was not available, although sensitivity analyses restricted to patients with persistent BB use and persistent non-use yielded findings consistent with the primary analysis. Finally, as with all registry-based studies, causal inferences should be drawn cautiously, and findings may not generalize to settings with different patient populations or infrastructure.

## Conclusions

This large, multicentre, real-world analysis did not identify a clear association between BB prescription at discharge and reduced long-term all-cause mortality after ACS. Although the largest short-term survival differences were observed among patients with reduced LVEF, uncertainty increased over longer follow-up. These findings add to the ongoing debate regarding the routine long-term use of BB therapy in contemporary ACS populations and highlight the need for further evaluation of patient selection and treatment duration.

## Disclaimers

None.

## Supplementary Material

oeag175_Supplementary_Data

## Data Availability

Anonymized data may be made available from the corresponding author upon reasonable request.

## Trial registration

This was an observational analysis using data from the CardioMining study, registered on ClinicalTrials.gov (Identifier: NCT05176769).
