# Supplementary material for: Beta-blockers after acute coronary syndrome: insights across the ejection fraction spectrum
Source: Eur Heart J Open. 2026 Oct 14;6(6):oeag175. doi: 10.1093/ehjopen/oeag175 (PMC13732307; doi:10.1093/ehjopen/oeag175)
Supplement: Supplementary file 1 [file oeag175_supplementary_data.docx]

**Beta-blockers after acute coronary syndrome: insights across the ejection fraction spectrum**

Eirinaios Tsiartas, Sokratis Oikonomou, Aristea Iordanidou, Athanasios Samaras, Andreas S Papazoglou, Dimitrios V Moysidis, Vassileios Vassilikos, Antonios Ziakas, Periklis Davlouros, Konstantinos Tsioufis, Dimitrios N Tziakas, Ioannis Zarifis, George Giannakoulas

# **Supplementary appendix**

| **Page** | **Supplementary item** |
| --- | --- |
| **2** | **Table S1:** STROBE checklist |
| **5** | **Table S2:** Contribution of participating hospitals to the CardioMining database and the study cohort |
| **6** | **Table S3:** Invasive management and reperfusion according to ACS presentation in the study cohort. |
| **7** | **Table S4:** Distribution of beta-blocker agents overall and according to LVEF category |
| **8** | **Table S5:** Comparison of included and excluded patient populations |
| **9** | **Table S6:** Propensity score model diagnostics and weight distribution |
| **10** | **Table S7:** Baseline characteristics after IPTW weighting according to discharge beta-blocker prescription |
| **11** | **Table S8:** IPTW-weighted restricted mean survival time and survival probability according to discharge beta-blocker prescription |
| **12** | **Table S9:** Association between clinical variables and all-cause mortality after acute coronary syndrome (supportive IPTW-weighted Cox model) |
| **13** | **Table S10:** Proportional hazards assumption testing using Schoenfeld residuals (supportive IPTW-weighted Cox model) |
| **14** | **Table S11:** Unweighted restricted mean survival time and survival probability according to discharge beta-blocker prescription |
| **15** | **Table S12:** IPTW-weighted restricted mean survival time and survival probability differences according to PCI status and ACS subtype |
| **16** | **Table S13:** Persistent beta-blocker use sensitivity analysis according to LVEF category |
| **17** | **Figure S1:** Standardised mean differences before and after inverse probability of treatment weighting |

**Table S1:** STROBE checklist

|  | **Item Description** | Location (or reason for not reporting) |
| --- | --- | --- |
| **Title and abstract** | | |
| [1a. Indicate the study’s design](https:/resources.equator-network.org/reporting-guidelines/strobe/items/title-abstract-indicate-study-design.html?utm_source=strobe&utm_medium=checklist&utm_campaign=1_1) | Indicate the study’s design with a commonly used term in the title or the abstract. | Abstract, methods paragraph. |
| [1b. Abstract](https:/resources.equator-network.org/reporting-guidelines/strobe/items/abstract.html?utm_source=strobe&utm_medium=checklist&utm_campaign=1_1) | Provide in the abstract an informative and balanced summary of what was done and what was found. | Abstract, methods and results paragraphs. |
| **Introduction** | | |
| [2. Background / rationale](https:/resources.equator-network.org/reporting-guidelines/strobe/items/background-rationale.html?utm_source=strobe&utm_medium=checklist&utm_campaign=1_1) | Explain the scientific background and rationale for the investigation being reported. | Introduction, paragraphs 1+2 |
| [3. Objectives](https:/resources.equator-network.org/reporting-guidelines/strobe/items/objectives.html?utm_source=strobe&utm_medium=checklist&utm_campaign=1_1) | State specific objectives, including any prespecified hypotheses. | Introduction, paragraph 3 |
| **Methods** | | |
| [4. Study design](https:/resources.equator-network.org/reporting-guidelines/strobe/items/study-design.html?utm_source=strobe&utm_medium=checklist&utm_campaign=1_1) | Present key elements of study design early in the paper. | Methods, study design paragraph |
| [5. Setting](https:/resources.equator-network.org/reporting-guidelines/strobe/items/setting.html?utm_source=strobe&utm_medium=checklist&utm_campaign=1_1) | Describe the setting, locations, and relevant dates, including periods of recruitment, exposure, follow-up, and data collection. | Methods, Population, data collection, and outcome of interest paragraph |
| [6a. Eligibility criteria](https:/resources.equator-network.org/reporting-guidelines/strobe/items/eligibility-criteria.html?utm_source=strobe&utm_medium=checklist&utm_campaign=1_1) | **Cohort study:** Give the eligibility criteria, and the sources and methods of selection of participants. Describe methods of follow-up. **Case-control study:** Give the eligibility criteria, and the sources and methods of case ascertainment and control selection. Give the rationale for the choice of cases and controls. **Cross-sectional study:** Give the eligibility criteria, and the sources and methods of selection of participants. | Methods, Population, data collection, and outcome of interest paragraph |
| [6b. Matching criteria](https:/resources.equator-network.org/reporting-guidelines/strobe/items/matching-criteria.html?utm_source=strobe&utm_medium=checklist&utm_campaign=1_1) | **Cohort study:** For matched studies, give matching criteria and number of exposed and unexposed. **Case-control study:** For matched studies, give matching criteria and the number of controls per case. | Methods, statistical analysis paragraph (for weighted analysis, as not matched study) |
| [7. Variables](https:/resources.equator-network.org/reporting-guidelines/strobe/items/variables.html?utm_source=strobe&utm_medium=checklist&utm_campaign=1_1) | Clearly define all outcomes, exposures, predictors, potential confounders, and effect modifiers. Give diagnostic criteria, if applicable. | Methods, statistical analysis paragraph |
| [8. Data sources / measurement](https:/resources.equator-network.org/reporting-guidelines/strobe/items/data-sources-measurement.html?utm_source=strobe&utm_medium=checklist&utm_campaign=1_1) | For each variable of interest give sources of data and details of methods of assessment (measurement). Describe comparability of assessment methods if there is more than one group. | Methods, statistical analysis paragraph |
| [9. Bias](https:/resources.equator-network.org/reporting-guidelines/strobe/items/bias.html?utm_source=strobe&utm_medium=checklist&utm_campaign=1_1) | Describe any efforts to address potential sources of bias. | Methods, statistical analysis paragraph (additional analyses sub-paragraph) |
| [10. Study size](https:/resources.equator-network.org/reporting-guidelines/strobe/items/study-size.html?utm_source=strobe&utm_medium=checklist&utm_campaign=1_1) | Explain how the study size was arrived at. | Methods, Population, data collection, and outcome of interest paragraph |
| [11. Quantitative variables](https:/resources.equator-network.org/reporting-guidelines/strobe/items/quantitative-variables.html?utm_source=strobe&utm_medium=checklist&utm_campaign=1_1) | Explain how quantitative variables were handled in the analyses. If applicable, describe which groupings were chosen, and why. | Methods, statistical analysis paragraph |
| [12a. Statistical methods](https:/resources.equator-network.org/reporting-guidelines/strobe/items/statistical-methods-description.html?utm_source=strobe&utm_medium=checklist&utm_campaign=1_1) | Describe all statistical methods, including those used to control for confounding. | Methods, statistical analysis paragraph |
| [12b. Statistical methods – subgroups and interactions](https:/resources.equator-network.org/reporting-guidelines/strobe/items/statistical-methods-subgroups-interactions.html?utm_source=strobe&utm_medium=checklist&utm_campaign=1_1) | Describe any methods used to examine subgroups and interactions. | Methods, statistical analysis paragraph |
| [12c. Statistical methods – missing data](https:/resources.equator-network.org/reporting-guidelines/strobe/items/statistical-methods-missing-data.html?utm_source=strobe&utm_medium=checklist&utm_campaign=1_1) | Explain how missing data were addressed. | Methods, statistical analysis paragraph |
| [12di. Statistical methods – loss to follow-up](https:/resources.equator-network.org/reporting-guidelines/strobe/items/statistical-methods-loss-to-follow-up.html?utm_source=strobe&utm_medium=checklist&utm_campaign=1_1) | **Cohort study:** If applicable, describe how loss to follow-up was addressed. | Methods, statistical analysis paragraph |
| [12dii. Statistical methods – matching cases and controls](https:/resources.equator-network.org/reporting-guidelines/strobe/items/statistical-methods-matching-cases-controls.html?utm_source=strobe&utm_medium=checklist&utm_campaign=1_1) | **Case-control study:** If applicable, explain how matching of cases and controls was addressed. | n/a – cohort study |
| [12diii. Statistical methods – sampling strategy](https:/resources.equator-network.org/reporting-guidelines/strobe/items/statistical-methods-analytical-methods-sampling-strategy.html?utm_source=strobe&utm_medium=checklist&utm_campaign=1_1) | **Cross-sectional study:** If applicable, describe analytical methods taking account of sampling strategy. | n/a – cohort study |
| [12e. Statistical methods – sensitivity analyses](https:/resources.equator-network.org/reporting-guidelines/strobe/items/statistical-methods-sensitivity-analyses.html?utm_source=strobe&utm_medium=checklist&utm_campaign=1_1) | Describe any sensitivity analyses. | Methods, statistical analysis paragraph (additional analyses sub-paragraph) |
| **Results** | | |
| [13a. Participant numbers](https:/resources.equator-network.org/reporting-guidelines/strobe/items/participants-numbers.html?utm_source=strobe&utm_medium=checklist&utm_campaign=1_1) | Report the numbers of individuals at each stage of the study—e.g., numbers potentially eligible, examined for eligibility, confirmed eligible, included in the study, completing follow-up, and analysed; Consider use of a flow diagram. | Results, patient characteristics paragraph and Figure 1 |
| [13b. Participants – non-participation](https:/resources.equator-network.org/reporting-guidelines/strobe/items/participants-non-participation.html?utm_source=strobe&utm_medium=checklist&utm_campaign=1_1) | Give reasons for non-participation at each stage. | Figure 1 |
| [13c. Participants – flow diagram](https:/resources.equator-network.org/reporting-guidelines/strobe/items/participants-flow-diagram.html?utm_source=strobe&utm_medium=checklist&utm_campaign=1_1) | Consider use of a flow diagram. | Figure 1 |
| [14a. Descriptive data – participant characteristics](https:/resources.equator-network.org/reporting-guidelines/strobe/items/descriptive-data-participant-characteristics.html?utm_source=strobe&utm_medium=checklist&utm_campaign=1_1) | Give characteristics of study participants (e.g., demographic, clinical, social) and information on exposures and potential confounders. Present the information in a table. | Results, patient characteristics paragraph and Tables 1 + S4 |
| [14b. Descriptive data – missing data](https:/resources.equator-network.org/reporting-guidelines/strobe/items/descriptive-data-missing-data.html?utm_source=strobe&utm_medium=checklist&utm_campaign=1_1) | Indicate the number of participants with missing data for each variable of interest. | Figure 1, Tables 1+2 |
| [14c. Descriptive data – follow-up time](https:/resources.equator-network.org/reporting-guidelines/strobe/items/descriptive-data-follow-up-time.html?utm_source=strobe&utm_medium=checklist&utm_campaign=1_1) | **Cohort study:** Summarise follow-up time—e.g., average and total amount. | Results, patient characteristics paragraph |
| [15. Outcome data](https:/resources.equator-network.org/reporting-guidelines/strobe/items/outcome-data.html?utm_source=strobe&utm_medium=checklist&utm_campaign=1_1) | **Cohort study:** Report numbers of outcome events or summary measures over time. **Case-control study:** Report numbers in each exposure category, or summary measures of exposure. **Cross-sectional study:** Report numbers of outcome events or summary measures. | Results, patient characteristics paragraph |
| [16a. Main results](https:/resources.equator-network.org/reporting-guidelines/strobe/items/main-results.html?utm_source=strobe&utm_medium=checklist&utm_campaign=1_1) | Give unadjusted estimates and, if applicable, confounder-adjusted estimates and their precision (e.g., 95% confidence intervals). Make clear which confounders were adjusted for and why they were included. | Results, Association between discharge BB prescription and survival |
| [16b. Main results – category boundaries](https:/resources.equator-network.org/reporting-guidelines/strobe/items/main-results-category-boundaries.html?utm_source=strobe&utm_medium=checklist&utm_campaign=1_1) | Report category boundaries when continuous variables were categorised. | Results, Tables 1 + S7 |
| [16c. Main results – risk](https:/resources.equator-network.org/reporting-guidelines/strobe/items/main-results-risk.html?utm_source=strobe&utm_medium=checklist&utm_campaign=1_1) | If relevant, consider translating estimates of relative risk into absolute risk for a meaningful time period. | Results, Reported as absolute RMST and survival probability differences |
| [17. Other analyses](https:/resources.equator-network.org/reporting-guidelines/strobe/items/other-analyses.html?utm_source=strobe&utm_medium=checklist&utm_campaign=1_1) | Report other analyses done—e.g., analyses of subgroups and interactions, and sensitivity analyses. | Results, Association according to PCI status and ACS subtype + Persistent BB use/non-use sensitivity analysis |
| **Discussion** |  |  |
| [18. Key results](https:/resources.equator-network.org/reporting-guidelines/strobe/items/key-results.html?utm_source=strobe&utm_medium=checklist&utm_campaign=1_1) | Summarise key results with reference to study objectives. | Discussion, paragraph 1 |
| [19. Limitations](https:/resources.equator-network.org/reporting-guidelines/strobe/items/limitations.html?utm_source=strobe&utm_medium=checklist&utm_campaign=1_1) | Discuss limitations of the study, taking into account sources of potential bias or imprecision. Discuss both direction and magnitude of any potential bias. | Discussion, paragraph 5 |
| [20. Interpretation](https:/resources.equator-network.org/reporting-guidelines/strobe/items/interpretation.html?utm_source=strobe&utm_medium=checklist&utm_campaign=1_1) | Give a cautious overall interpretation considering objectives, limitations, multiplicity of analyses, results from similar studies, and other relevant evidence. | Discussion, paragraphs 2-4 |
| [21. Generalisability](https:/resources.equator-network.org/reporting-guidelines/strobe/items/generalisability.html?utm_source=strobe&utm_medium=checklist&utm_campaign=1_1) | Discuss the generalisability (external validity) of the study results. | Discussion, paragraphs 2-4 |
| **Other information** |  |  |
| [22. Funding](https:/resources.equator-network.org/reporting-guidelines/strobe/items/funding.html?utm_source=strobe&utm_medium=checklist&utm_campaign=1_1) | Give the source of funding and the role of the funders for the present study and, if applicable, for the original study on which the present article is based. | Declarations, Funding (no external funding received) |

**Table S2:** Contribution of participating hospitals to the CardioMining database and the study cohort.

| **Hospital** | **Total cardiovascular hospitalisations in CardioMining** | **ACS admissions in registry** | **Included in final cohort, n (% of ACS admissions)** | **Years contributed** |
| --- | --- | --- | --- | --- |
| AHEPA University Hospital, Thessaloniki | 9176 | 2,294 | 2,220 (96.8%) | 2013–2019 |
| General Hospital of Thessaloniki "Papanikolaou", Thessaloniki | 6561 | 2,241 | 2,162 (96.5%) | 2015–2023 |
| University General Hospital of Alexandroupolis, Alexandroupolis | 7451 | 1,677 | 626 (37.3%) | 2010–2021 |
| Hippokration General Hospital of Athens, Athens | 2887 | 720 | 597 (82.9%) | 2018–2023 |
| University General Hospital of Patras, Patras | 14395 | 5,620 | 0 | 2016–2021 |
| Korgialenio-Benakio Hellenic Red Cross Hospital, Athens | 2926 | 622 | 0 | 2016–2022 |
| Hippokration General Hospital of Thessaloniki, Thessaloniki | 2909 | 627 | 444 (70.8%) | 2015–2023 |

*ACS, acute coronary syndrome; LVEF, left ventricular ejection fraction.*

Note: Eligibility for analysis required both baseline LVEF data and successful linkage to national mortality records using patient social security identifiers. Availability of linkage data varied across centres and calendar years because the national electronic prescribing system used for mortality ascertainment was introduced in late 2011 and adopted progressively thereafter. Hospitals 5 and 6 did not provide mortality linkage data and were therefore excluded from the mortality analyses despite contributing ACS admissions to the registry.

**Table S3:** Invasive management and reperfusion according to ACS presentation in the study cohort.

| **ACS presentation** | **N** | **Coronary angiography, n (%)** | **PCI, n (%)** | **Thrombolysis, n (%)** |
| --- | --- | --- | --- | --- |
| STEMI | 2,165 | 1,772 (81.8%) | 1,436 (66.3%) | 317 (14.6%) |
| NSTEMI | 2,304 | 1,416 (61.5%) | 837 (36.3%) | 36 (1.6%) |
| Unstable angina | 1,580 | 1,059 (67.0%) | 652 (41.3%) | 48 (3.0%) |

**Table S4:** Distribution of beta-blocker agents overall and according to LVEF category

| **Agent** | **Overall** | **LVEF ≤40%** | **LVEF 41–49%** | **LVEF ≥50%** |
| --- | --- | --- | --- | --- |
| Bisoprolol | 2,239 (43.8%) | 619 (48.5%) | 299 (49.0%) | 1,321 (41.0%) |
| Metoprolol | 1,574 (30.8%) | 240 (18.8%) | 195 (32.0%) | 1,139 (35.4%) |
| Carvedilol | 982 (19.2%) | 364 (28.5%) | 88 (14.4%) | 530 (16.5%) |
| Nebivolol | 268 (5.2%) | 44 (3.4%) | 26 (4.3%) | 198 (6.1%) |
| Sotalol | 38 (0.7%) | 8 (0.6%) | 1 (0.2%) | 29 (0.9%) |
| Propranolol | 6 (0.1%) | 2 (0.2%) | 1 (0.2%) | 3 (0.1%) |

*LVEF, left ventricular ejection fraction*.

Note: Sotalol was included in the beta-blocker category because of its beta-blocking activity and represented only 0.7% of prescriptions.

**Table S5:** Comparison of included and excluded (due to loss to follow-up or no LVEF measurement availability) patient populations

|  | **Overall** | **Excluded** | **Included** |
| --- | --- | --- | --- |
| **n** | 13801 | 7752 | 6049 |
| **Age** | 66.0 (±12.4) | 65.3 (±11.5) | 66.8 (±13.2) |
| **LVEF** | 53.7 (±14.8) | 53.4 (±13.5) | 54.0 (±16.1) |
| **Male** | 10541 (76.4%) | 6008 (77.5%) | 4533 (74.9%) |
| **ACS type:**  **-STEMI**  **-NSTEMI**  **-Unstable angina** | 5082 (36.8%)  5747 (41.6%)  2972 (21.5%) | 2917 (37.6%)  3443 (44.4%)  1392 (18.0%) | 2165 (35.8%)  2304 (38.1%)  1580 (26.1%) |
|  |  |  |  |
|  |  |  |  |
| **PCI during admission** | 6865 (49.7%) | 3940 (50.8%) | 2925 (48.4%) |
| **Invasive angiography during admission** | 10512 (76.2%) | 6265 (80.8%) | 4247 (70.2%) |
| **Thrombolysis** | 808 (5.9%) | 407 (5.3%) | 401 (6.6%) |
| **Current smoker** | 4965 (36.0%) | 2943 (38.0%) | 2022 (33.4%) |
| **Hypertension** | 6699 (48.5%) | 3863 (49.8%) | 2836 (46.9%) |
| **Dyslipidaemia** | 4138 (30.0%) | 2739 (35.3%) | 1399 (23.1%) |
| **Type 2 diabetes mellitus** | 3782 (27.4%) | 2017 (26.0%) | 1765 (29.2%) |
| **COPD** | 673 (4.9%) | 410 (5.3%) | 263 (4.3%) |
| **CKD** | 913 (6.6%) | 365 (4.7%) | 548 (9.1%) |
| **Carotid artery disease** | 217 (1.6%) | 130 (1.7%) | 87 (1.4%) |
| **Peripheral artery disease** | 371 (2.7%) | 216 (2.8%) | 155 (2.6%) |
| **Heart failure** | 778 (5.6%) | 319 (4.1%) | 459 (7.6%) |
| **Prior CAD** | 5524 (40.0%) | 2989 (38.6%) | 2535 (41.9%) |
| **Prior ACS** | 3373 (24.4%) | 1917 (24.7%) | 1456 (24.1%) |
| **LVEF≤40%** | 2766 (21.9%) | 1289 (19.5%) | 1477 (24.4%) |

*ACS, acute coronary syndrome; CAD, coronary artery disease; CKD, chronic kidney disease; COPD, chronic obstructive pulmonary disease; LVEF, left ventricular ejection fraction; NSTEMI, non-ST elevation myocardial infarction; PCI, percutaneous coronary intervention; STEMI, ST elevation myocardial infarction.*

Note: Excluded patients comprised all individuals not meeting the inclusion criteria because of unavailable follow-up data and/or missing LVEF measurements. Percentages for the LVEF≤40% category were calculated among patients with available LVEF measurements within each column. LVEF data were available for 12,658 patients overall, 6,609 excluded patients, and 6,049 included patients.

**Table S6:** Propensity score model diagnostics and weight distribution

| **Metric** | **Value** |
| --- | --- |
| Patients receiving discharge beta-blockers | 5,107 |
| Patients not receiving discharge beta-blockers | 942 |
| Propensity score range | 0.594–0.954 |
| Median propensity score | 0.851 |
| Mean stabilised weight | 1.000 |
| Median stabilised weight | 0.988 |
| Stabilised weight range before truncation | 0.472–2.273 |
| Stabilised weight range after truncation | 0.629–1.495 |
| Patients truncated at lower bound (1st percentile) | 61 |
| Patients truncated at upper bound (99th percentile) | 61 |
| Effective sample size, beta-blocker group | 5,091 |
| Effective sample size, no beta-blocker group | 885 |
| Propensity model C-statistic | 0.599 |

**Table S7:** Baseline characteristics after IPTW weighting according to discharge beta-blocker prescription.

| **Characteristic** | **No BB (weighted n=933.6)** | **BB (weighted n=5107.4)** | **SMD** |
| --- | --- | --- | --- |
| Age, years | 67.09 ± 13.61 | 66.78 ± 13.15 | 0.023 |
| Male sex | 698.0 (74.8%) | 3827.2 (74.9%) | 0.004 |
| Follow-up duration, years | 4.8 (2.4–7.0%) | 4.5 (2.4–6.4%) | 0.102 |
| All-cause mortality | 295.9 (31.7%) | 1619.8 (31.7%) | 0.001 |
| **ACS presentation** | | | |
| STEMI | 329.1 (35.3%) | 1826.8 (35.8%) | 0.014 |
| NSTEMI | 355.5 (38.1%) | 1946.2 (38.1%) |  |
| Unstable angina | 249.0 (26.7%) | 1334.4 (26.1%) |  |
| PCI during admission | 446.1 (47.8%) | 2469.4 (48.4%) | 0.011 |
| **LVEF** | | | |
| LVEF numerical | 54.32 ± 16.17 | 54.02 ± 16.20 | 0.018 |
| ≤40% | 222.5 (23.8%) | 1253.6 (24.5%) | 0.098 |
| 41–49% | 83.5 (8.9%) | 599.1 (11.7%) |  |
| ≥50% | 627.5 (67.2%) | 3254.7 (63.7%) |  |
| **Prior history** | | | |
| Previous CAD | 398.1 (42.6%) | 2142.0 (41.9%) | 0.014 |
| Previous heart failure | 70.8 (7.6%) | 387.7 (7.6%) | <0.001 |
| Hypertension | 437.9 (46.9%) | 2394.6 (46.9%) | <0.001 |
| Type 2 diabetes mellitus | 268.0 (28.7%) | 1489.8 (29.2%) | 0.010 |
| Chronic kidney disease | 87.1 (9.3%) | 463.1 (9.1%) | 0.009 |
| Chronic obstructive pulmonary disease | 42.7 (4.6%) | 222.6 (4.4%) | 0.010 |
| Atrial fibrillation | 89.2 (9.6%) | 465.9 (9.1%) | 0.015 |

*ACS: acute coronary syndrome; BB: beta blocker; CAD: coronary artery disease; LVEF: left ventricular ejection fraction; NSTEMI: non-ST elevation myocardial infarction; PCI: percutaneous coronary intervention; STEMI: ST elevation myocardial infarction.*

**Table S8:** IPTW-weighted restricted mean survival time and survival probability according to discharge beta-blocker prescription

| **No BB RMST** | **BB RMST** | **RMST difference (95% CI), days** | **No BB survival** | **BB survival** | **Survival difference (95% CI)** |
| --- | --- | --- | --- | --- | --- |
| **Overall** | | | | | |
| *1 year* | | | | | |
| 331.5 | 337.9 | 6.3 (-0.0 to 13.2) | 0.870 | 0.891 | 0.021 (-0.001 to 0.042) |
| *5 years* | | | | | |
| 1484.7 | 1496 | 11.3 (-30.7 to 63.9) | 0.718 | 0.713 | -0.005 (-0.034 to 0.032) |
| **LVEF ≤40%** | | | | | |
| *1 year* | | | | | |
| 291.5 | 310.3 | 18.8 (1.1 to 37.4) | 0.726 | 0.781 | 0.055 (0.000 to 0.119) |
| *5 years* | | | | | |
| 1156.8 | 1208.9 | 52.1 (-56.5 to 179.2) | 0.497 | 0.494 | -0.003 (-0.074 to 0.098) |
| **LVEF 41–49%** | | | | | |
| *1 year* | | | | | |
| 330.4 | 335.9 | 5.5 (-13.2 to 27.4) | 0.866 | 0.882 | 0.015 (-0.052 to 0.089) |
| *5 years* | | | | | |
| 1447.8 | 1482.3 | 34.5 (-106.6 to 183.2) | 0.695 | 0.701 | 0.006 (-0.080 to 0.112) |
| **LVEF ≥50%** | | | | | |
| *1 year* | | | | | |
| 345.9 | 348.8 | 2.9 (-2.3 to 9.3) | 0.922 | 0.935 | 0.013 (-0.007 to 0.029) |
| *5 years* | | | | | |
| 1607.1 | 1608.5 | 1.4 (-38.7 to 38.8) | 0.800 | 0.798 | -0.002 (-0.033 to 0.036) |

*BB: beta-blocker; CI: confidence interval; IPTW: inverse probability of treatment weighting; LVEF: left ventricular ejection fraction; RMST: restricted mean survival time.*

**Table S9:** Association between clinical variables and all-cause mortality after acute coronary syndrome (supportive IPTW-weighted Cox model)

| **Variable** | **aHR (95% CI)** | **p-value** |
| --- | --- | --- |
| **Discharge BB prescription in LVEF ≤40% (reference)** | 1.07 (0.83–1.38) | 0.602 |
| **BB × LVEF interaction** | | 0.967 (global p) |
| BB association in LVEF 41–49% relative to ≤40% | 1.03 (0.63–1.67) | 0.920 |
| BB association in LVEF ≥50% relative to ≤40% | 0.97 (0.72–1.32) | 0.865 |
| **ACS subtype** |  |  |
| STEMI | Reference |  |
| NSTEMI | 1.32 (1.17–1.49) | <0.001 |
| Unstable angina | 1.10 (0.96–1.26) | 0.163 |
| **LVEF category** |  |  |
| ≤40% | Reference |  |
| 41–49% | 0.73 (0.46–1.15) | 0.178 |
| ≥50% | 0.53 (0.40–0.71) | <0.001 |
| **Other predictors** |  |  |
| Age (per year) | 1.05 (1.05–1.06) | <0.001 |
| Male sex | 0.88 (0.79–0.98) | 0.020 |
| PCI during admission | 0.67 (0.60–0.74) | <0.001 |
| Prior CAD | 1.17 (1.05–1.29) | 0.003 |
| Heart failure | 1.09 (0.92–1.29) | 0.330 |
| Hypertension | 0.89 (0.81–0.98) | 0.021 |
| T2DM | 1.38 (1.25–1.52) | <0.001 |
| Chronic kidney disease | 1.88 (1.63–2.17) | <0.001 |
| COPD | 1.66 (1.38–2.01) | <0.001 |
| Atrial fibrillation | 1.21 (1.05–1.39) | 0.009 |

*ACS: acute coronary syndrome; aHR: adjusted hazard ratio; BB: beta-blocker; CAD: coronary artery disease; CI: confidence interval; COPD: chronic obstructive pulmonary disease; IPTW: inverse probability of treatment weighting; LVEF: left ventricular ejection fraction; NSTEMI: non-ST-elevation myocardial infarction; PCI: percutaneous coronary intervention; STEMI: ST-elevation myocardial infarction; T2DM: type 2 diabetes mellitus.*

**Table S10:** Proportional hazards assumption testing using Schoenfeld residuals **(**supportive IPTW-weighted Cox model)

| **Variable** | **χ²** | **df** | **p-value** |
| --- | --- | --- | --- |
| Discharge BB prescription | 5.58 | 1 | 0.018 |
| BB × LVEF category interaction | 11.09 | 2 | 0.004 |
| ACS subtype | 3.99 | 2 | 0.136 |
| LVEF category | 13.17 | 2 | 0.001 |
| Age | 0.88 | 1 | 0.349 |
| Male sex | 5.26 | 1 | 0.022 |
| PCI during admission | 25.29 | 1 | <0.001 |
| Prior CAD | 3.34 | 1 | 0.068 |
| Heart failure | 3.74 | 1 | 0.053 |
| Hypertension | 0.90 | 1 | 0.343 |
| Diabetes mellitus | 0.07 | 1 | 0.789 |
| Chronic kidney disease | 0.81 | 1 | 0.367 |
| COPD | <0.001 | 1 | 0.997 |
| Atrial fibrillation | 0.73 | 1 | 0.393 |
| **Global test** | **63.54** | **17** | **<0.001** |

*ACS: acute coronary syndrome; BB: beta-blocker; CAD: coronary artery disease; COPD: chronic obstructive pulmonary disease; df: degrees of freedom; LVEF: left ventricular ejection fraction; PCI: percutaneous coronary intervention.*

**Table S11:** Unweighted restricted mean survival time and survival probability according to discharge beta-blocker prescription

| **No BB RMST** | **BB RMST** | **RMST difference (95% CI), days** | **No BB survival** | **BB survival** | **Survival difference (95% CI)** |
| --- | --- | --- | --- | --- | --- |
| **Overall** | | | | | |
| *1 year* | | | | | |
| 329.3 | 338.3 | 9.0 (1.1 to 15.1) | 0.862 | 0.893 | 0.031 (0.006 to 0.053) |
| *5 years* | | | | | |
| 1463.8 | 1501.2 | 37.4 (-7.4 to 80.9) | 0.702 | 0.717 | 0.015 (-0.015 to 0.043) |
| **LVEF ≤40%** | | | | | |
| *1 year* | | | | | |
| 287.7 | 311.2 | 23.5 (5.5 to 44.4) | 0.710 | 0.785 | 0.075 (0.009 to 0.149) |
| *5 years* | | | | | |
| 1125.0 | 1216.6 | 91.6 (-24.1 to 218.8) | 0.476 | 0.500 | 0.024 (-0.056 to 0.109) |
| **LVEF 41–49%** | | | | | |
| *1 year* | | | | | |
| 320.5 | 336.9 | 16.4 (-6.1 to 44.2) | 0.837 | 0.886 | 0.050 (-0.020 to 0.125) |
| *5 years* | | | | | |
| 1381.3 | 1493.2 | 111.9 (-60.0 to 256.2) | 0.652 | 0.708 | 0.057 (-0.068 to 0.164) |
| **LVEF ≥50%** | | | | | |
| *1 year* | | | | | |
| 343.0 | 349.4 | 6.4 (0.1 to 13.5) | 0.912 | 0.937 | 0.025 (-0.001 to 0.049) |
| *5 years* | | | | | |
| 1577.2 | 1615.0 | 37.8 (-6.1 to 78.9) | 0.777 | 0.803 | 0.027 (-0.015 to 0.062) |

*BB: beta-blocker; CI: confidence interval; IPTW: inverse probability of treatment weighting; LVEF: left ventricular ejection fraction; RMST: restricted mean survival time.*

**Table S12:** IPTW-weighted restricted mean survival time and survival probability differences according to PCI status and ACS subtype

| **No BB RMST** | **BB RMST** | **RMST difference (95% CI), days** | **No BB survival** | **BB survival** | **Survival difference (95% CI)** |
| --- | --- | --- | --- | --- | --- |
| **PCI during index admission analysis** | | | | | |
| **PCI** | | | | | |
| *1 year* | | | | | |
| 353.3 | 353.9 | 0.6 (-5.1 to 6.0) | 0.950 | 0.956 | 0.006 (-0.017 to 0.024) |
| *5 years* | | | | | |
| 1678.2 | 1662.9 | -15.3 (-61.5 to 30.3) | 0.857 | 0.838 | -0.019 (-0.062 to 0.029) |
| **No PCI** | | | | | |
| *1 year* | | | | | |
| 311.6 | 322.8 | 11.3 (-0.0 to 22.6) | 0.797 | 0.830 | 0.034 (-0.002 to 0.068) |
| *5 years* | | | | | |
| 1306.3 | 1337.7 | 31.4 (-32.3 to 101.3) | 0.588 | 0.592 | 0.004 (-0.038 to 0.052) |
| **ACS subtype analysis** | | | | | |
| **STEMI** | | | | | |
| *1 year* | | | | | |
| 337.1 | 348.4 | 11.3 (4.0 to 22.2) | 0.912 | 0.937 | 0.025 (0.003 to 0.056) |
| *5 years* | | | | | |
| 1580.4 | 1607.2 | 26.8 (-20.6 to 80.3) | 0.790 | 0.802 | 0.012 (-0.031 to 0.057) |
| **NSTEMI** | | | | | |
| *1 year* | | | | | |
| 321.5 | 324.7 | 3.2 (-7.6 to 15.2) | 0.817 | 0.836 | 0.019 (-0.023 to 0.067) |
| *5 years* | | | | | |
| 1344.8 | 1364.7 | 19.9 (-56.2 to 95.5) | 0.613 | 0.614 | 0.001 (-0.060 to 0.065) |
| **Unstable angina** | | | | | |
| *1 year* | | | | | |
| 338.5 | 342.6 | 4.1 (-7.5 to 13.8) | 0.891 | 0.909 | 0.019 (-0.019 to 0.050) |
| *5 years* | | | | | |
| 1559.5 | 1532.5 | -27.0 (-94.5 to 54.2) | 0.773 | 0.730 | -0.043 (-0.103 to 0.009) |

*ACS: acute coronary syndrome; BB: beta-blocker; CI: confidence interval; IPTW: inverse probability of treatment weighting; NSTEMI: non-ST elevation myocardial infarction; PCI: percutaneous coronary intervention; RMST: restricted mean survival time; STEMI: ST elevation myocardial infarction.*

**Table S13:** Persistent beta-blocker use sensitivity analysis according to LVEF category

| **Persistent BB non-use RMST** | **Persistent BB use RMST** | **RMST difference (95% CI), days** | **Persistent BB non-use survival** | **Persistent BB use survival** | **Survival difference (95% CI)** |
| --- | --- | --- | --- | --- | --- |
| **Overall** | | | | | |
| *1 year* | | | | | |
| 332.8 | 336 | 3.2 (-4.7 to 12.8) | 0.877 | 0.884 | 0.007 (-0.022 to 0.035) |
| *5 years* | | | | | |
| 1496.1 | 1486.8 | -9.3 (-62.7 to 44.7) | 0.727 | 0.711 | -0.016 (-0.056 to 0.029) |
| **LVEF≤40%** | | | | | |
| *1 year* | | | | | |
| 294.0 | 307.6 | 13.5 (-13.2 to 39.2) | 0.745 | 0.774 | 0.029 (-0.064 to 0.108) |
| *5 years* | | | | | |
| 1129.8 | 1198.9 | 69.1 (-45.8 to 253.7) | 0.446 | 0.493 | 0.047 (-0.040 to 0.172) |
| **LVEF 41–49%** | | | | | |
| *1 year* | | | | | |
| 324.9 | 334.2 | 9.2 (-16.5 to 38.1) | 0.864 | 0.878 | 0.014 (-0.075 to 0.132) |
| *5 years* | | | | | |
| 1386.5 | 1478.1 | 91.6 (-81.1 to 275.1) | 0.634 | 0.709 | 0.075 (-0.055 to 0.235) |
| **LVEF≥50%** | | | | | |
| *1 year* | | | | | |
| 344.8 | 348.1 | 3.3 (-3.2 to 11.9) | 0.917 | 0.931 | 0.014 (-0.011 to 0.044) |
| *5 years* | | | | | |
| 1614.1 | 1606.9 | -7.1 (-61.4 to 44.5) | 0.818 | 0.800 | -0.018 (-0.070 to 0.024) |

*BB: beta-blocker; CI: confidence interval; LVEF: left ventricular ejection fraction; RMST: restricted mean survival time.*

**Figure S1:** Standardised mean differences before and after inverse probability of treatment weighting.

*ACS, acute coronary syndrome; AF, atrial fibrillation; BB, beta-blocker; CAD, coronary artery disease; CKD, chronic kidney disease; COPD, chronic obstructive pulmonary disease; HF, heart failure; IPTW, inverse probability of treatment weighting; LVEF, left ventricular ejection fraction; NSTEMI, non-ST-elevation myocardial infarction; PCI, percutaneous coronary intervention; STEMI, ST-elevation myocardial infarction; T2DM, type 2 diabetes mellitus.*
